# The Efficacy of Ketamine for Acute and Chronic Pain in Patients with Cancer: A Systematic Review of Randomized Controlled Trials

**DOI:** 10.3390/healthcare12161560

**Published:** 2024-08-06

**Authors:** Leila Azari, Homa Hemati, Ronia Tavasolian, Sareh Shahdab, Stephanie M. Tomlinson, Margarita Bobonis Babilonia, Jeffrey Huang, Danielle B. Tometich, Kea Turner, Kimia Saleh Anaraki, Heather S. L. Jim, Amir Alishahi Tabriz

**Affiliations:** 1 Morsani College of Medicine, University of South Florida, Tampa, FL 33602, USA; smtomlinson@usf.edu; 2 College of Pharmacy, Tehran University of Medical Sciences, Tehran 1416753955, Iran; hemati.homa98@gmail.com (H.H.); shahdabsareh@gmail.com (S.S.); 3 Department of Clinical Science and Nutrition, University of Chester, Chester CH1 4BJ, UK; ronia_tsn@yahoo.com; 4 Supportive Care Medicine Department, Behavioral Medicine Services, Moffitt Cancer Center, Tampa, FL 33612, USA; margarita.bobonis-babilonia@va.gov; 5 Department of Psychiatry and Behavioral Medicine, University of South Florida Morsani College of Medicine, Tampa, FL 33602, USA; 6 Department of Oncological Sciences, University of South Florida Morsani College of Medicine, Tampa, FL 33602, USA; kea.turner@moffitt.org (K.T.); heather.jim@moffitt.org (H.S.L.J.); amir.alishahi@moffitt.org (A.A.T.); 7 Department of Anesthesiology, Moffitt Cancer Center, Tampa, FL 33612, USA; jeffrey.huang@moffitt.org; 8 Department of Health Outcomes and Behavior, Moffitt Cancer Center, Tampa, FL 33612, USA; danielle.tometich@moffitt.org; 9 Department of Internal Medicine, University of Maryland Capital Region, Largo, MD 20774, USA; kimia.salehanaraki@umm.edu

**Keywords:** ketamine, pain, cancer, oncology, refractory cancer pain

## Abstract

Managing cancer-related pain poses significant challenges, prompting research into alternative approaches such as ketamine. This systematic review aims to analyze and summarize the impact of ketamine as an adjuvant to opioid therapy for cancer-related pain. We conducted a literature review in MEDLINE, EMBASE, and Scopus from 1 January 1982 to 20 October 2023. Abstracts were screened against inclusion criteria, and eligible studies underwent a full-text review. Data was extracted from the included studies, and a framework analysis approach summarized the evidence regarding ketamine’s use in patients with cancer. A total of 21 randomized clinical trials were included, and the quality of all the included studies was good or fair. Significant improvements in pain scores and reduced morphine consumption were consistently observed with intravenous ketamine administration for postoperative pain control, particularly when combined with other analgesics such as morphine. Ketamine was less effective when used as an analgesic for chronic pain management, with several studies on neuropathic pain or chemotherapy-induced neuropathy finding minimal significant effect on reduction of pain scores or morphine requirements. The efficacy of ketamine in pain management appears to depend on factors such as dosage, route of administration, and patient population.

## 1. Introduction

Despite advancements in pharmacological therapies and understanding of the molecular mechanisms underlying cancer pain, the prevalence of cancer pain remains high [1]. Systematic reviews and meta-analyses reveal that over one-third of patients experience pain related to cancer after curative treatment, and two-thirds of patients with advanced or metastatic cancer report symptoms of pain [1,2]. Studies showed up to 20% of patients with cancer undergoing opioid titration develop refractory pain or experience a poor analgesic response and intolerable side effects [3,4]. Patients with cancer may also undergo a variety of surgeries, with pain being an expected outcome. Pain may develop because of the tumor itself, either through the obstruction of surrounding structures or invasion of tissue and subsequent inflammation [5]. Pre-existing oncological pain and opioid tolerance represent unique challenges in managing acute pain in the perioperative period in this specific population [6]. Additionally, chemotherapy-induced peripheral neuropathy (CIPN) is a common and challenging side effect associated with many anticancer agents that persists in 30% of patients following chemotherapy [7]. Inadequate pain management in cancer patients adversely affects physical function, compromises psychological well-being, disrupts social interactions, leads to increased emergency department visits and hospitalization, and undermines the effectiveness of antitumor treatment [8,9].

The management of moderate to severe cancer-related pain involves a combination of opioid analgesics administered in rotation and through dose titration to mitigate the effects of opioid toxicity [3,10,11,12]. The advent of stepwise multimodal approaches to pain management necessitates alternative therapeutic strategies beyond opioid administration to alleviate the symptoms associated with cancer-related pain. Ketamine, typically used as an anesthetic, is a *N*-methyl-D-aspartate (NMDA) antagonist capable of treating acute and chronic pain at low, subanesthetic doses [13]. Despite growing evidence regarding the benefits of ketamine as a rapid antidepressant and antisuicidal agent, [14] its efficacy in the treatment of chronic cancer pain remains unclear [15,16]. Ketamine can be administered as an adjuvant to opioid therapy in patients with cancer when their pain becomes opioid-resistant, improving patient outcomes and quality of life [4,17]. The various modes by which ketamine can be administered, along with variability in dosage and duration, pose a challenge in the creation of standardized treatment guidelines for this drug. Many clinicians may hesitate to administer ketamine considering the ambiguous clinical evidence and adverse event profile associated with the treatment [17].

This review aims to systematically summarize and analyze the existing literature on ketamine administration within the cancer population. The review specifically focuses on the effectiveness of ketamine as an adjuvant to opioid therapy for managing both acute and chronic pain among patients with cancer. Additionally, the review compares and reports different methods of ketamine administration in conjunction with opioids to identify optimal approaches for maximizing efficacy and minimizing side effects.

## 2. Materials and Methods

A systematic literature review was conducted according to Preferred Reporting Items for Systematic Reviews and Meta-Analyses guidelines (Appendix A). The study protocol was registered in the International Prospective Register of Systematic Reviews (PROSPERO) (registration number: CRD42022347551).

### 2.1. Study Inclusion and Exclusion Criteria

The inclusion criteria of the review consisted of articles assessing the relationship between administering ketamine to adult patients with cancer and pain. Additionally, articles were required to be peer-reviewed, report the results of a randomized controlled trial (RCT), and written in the English language. We decided to only include RCTs because systematic reviews of RCTs are regarded as the highest quality evidence [18,19]. Articles were excluded if the target population was children (younger than 18), in addition to any articles focused only on molecular aspects of ketamine. A detailed list of inclusion and exclusion criteria can be found in Appendix B.

### 2.2. Information Sources and Search Strategy

The initial search was intentionally broad to capture the inclusion criteria and to minimize the risk of overlooking potentially relevant studies. Cancer and ketamine administration were the main components of the search strategy. Using a combination of subject headings and keywords, the search strategy was implemented into MEDLINE^®^ (via PubMed^®^, Bethesda, MD, USA), EMBASE, and Scopus from 1 January 1982 to 20 October 2023, when all searches were completed. The citations of included studies for relevant articles and references were manually scanned from similar systematic reviews to ensure no relevant studies were missed during indexing. Gray literature was not included, as we considered only peer-reviewed published studies. To exclude animal studies, we applied the Cochrane human studies filter. We also added a systematic review keyword and publication type filter to exclude systematic review articles. Appendix C shows the complete strategy for each of the searches.

### 2.3. Study Selection Process

Two researchers screened the titles and abstracts against the eligibility criteria. Discrepancies were resolved through discussions between members of each pair. When necessary, a third team member reviewed the discrepancy until a consensus was reached. Inter-rater reliability of reviews was achieved by ensuring three iterations of sample reviews were conducted with each person reviewing 30 articles until an average agreement of 83% was reached. The full-text articles were screened in a similar manner.

### 2.4. Study Quality Assessment

Two independent researchers assessed the quality of included studies using the NIH Quality Assessment Tool for the controlled intervention studies [20]. We assigned the quality of each study as good, fair, or poor (see Appendix D), and any disagreements in the risk of bias scoring were resolved by consensus or by a discussion with a third author.

### 2.5. Data Extraction and Analysis

A meta-analysis was not conducted due to heterogeneity in populations and in how pain was measured. Using a framework analysis approach, we summarized the evidence on using ketamine in patients with cancer [21]. The framework analysis approach consisted of five stages: familiarization, framework selection, indexing, charting, and mapping and interpretation.

First, team members familiarized themselves with the literature in addition to reading included studies. Second, conceptual frameworks were identified that served as the codes for data abstraction. We used a thematic framework to describe studies in which research has investigated administering ketamine in patients with cancer, which included: publication year, design, outcome(s), type of cancer, objective(s), country, setting, dosage, outcomes, and the relationship between using the ketamine and outcomes. Data were also collected on the route of ketamine administration (e.g., infusion and intranasal). Pairs of authors completed charting and indexing by inputting selected text from included studies into the appropriate cells within our framework. Data extraction from the included studies was achieved using a standardized data extraction form in Microsoft Excel (version 2016). Last, extracted data were analyzed from each cell to describe the studies and findings of using ketamine in patients with cancer.

## 3. Results

### 3.1. Study Selection

**Figure 1:**
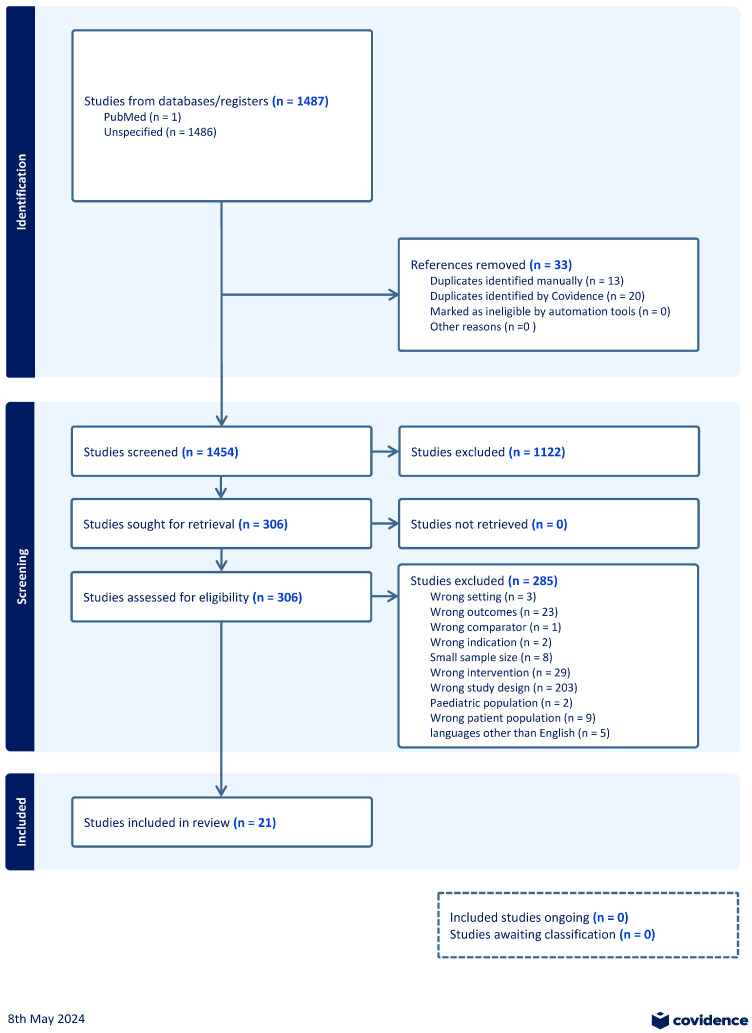
PRISMA flow diagram.

The searches in PubMed, Embase, and Scopus yielded 1487 citations. These citations were exported to Endnote (Version 20), and 33 duplicates were removed using the Endnote deduplication feature. This resulted in a total of 1454 unique citations found across all database searches. As can be seen in Figure 1, titles and abstracts of the 1454 articles were screened; 306 were selected for full-text screening. Of the 306 studies, 285 were excluded at full-text screening or during extraction attempts with the consensus of two co-authors; 21 unique eligible studies were included [22,23,24,25,26,27,28,29,30,31,32,33,34,35,36,37,38,39,40,41,42].

### 3.2. Characteristics of Included Studies

The included studies were published between 2001 and 2019. Included studies focused on different cancer types, including but not limited to abdominal cancer, breast cancer, lung cancer, colon cancer, and prostate cancer. Characteristics of included studies are shown in Table 1. We found that outcomes were primarily divided into two categories: treatment of pain postoperatively in patients with cancer undergoing oncologic surgery or treatment for refractory pain. Several studies also examined CIPN as a component of cancer-related pain. Ketamine was administered via various modes of delivery, including intrathecally, intramuscularly, subcutaneously, topically, orally, and intravenously.

### 3.3. Quality Assessment of Included Studies

The quality of all the included studies was good or fair. The details of the quality assessment of the included studies are shown in Appendix D.

### 3.4. Intravenous Administration of Ketamine

Eight studies examined the effect of intravenously administered ketamine on pain scores in patients with cancer [25,26,32,33,36,37,39,42]. While all eight studies examined pain as either a primary or secondary outcome, the type of pain assessed varied. Six studies investigated the effect of peri- or pre-operative ketamine on reducing postoperative pain scores following oncologic surgery in the inpatient setting [25,26,33,36,37,39], including pain at surgical sites [32], and one study focused on the use of ketamine for chronic pain therapy [42]. Further characteristics of the studies, such as the dosage and types of pain scores utilized, can be found in Table 2. Most studies examining postoperative outcomes used the visual analog scale (VAS) to evaluate pain scores [26,33,36,37,39,42]. Ketamine was used as the sole pharmacological treatment in only two of the eight studies [26,32]. Other studies compared the efficacy of morphine in combination with ketamine in reducing pain scores [25,33].

#### Effect on Pain Scores

Seven of the eight studies found significant improvement in pain scores following administration of ketamine [25,26,32,33,36,39,42]. However, while one study concluded that intraoperative infusions of ketamine helped postoperative pain up to three months after breast cancer surgery, it failed to reduce clinically significant pain and improve patients’ quality of life [32]. One of the eight studies did not find significant improvement in pain scores following administration of ketamine. The study found that IV ketamine administered throughout surgery reduced postoperative consumption of morphine but that there was no significant difference in VAS scores following surgery [37].

### 3.5. Intrathecal Administration of Ketamine

Three of the included studies examined outcomes of pain associated with intrathecal administration of ketamine hydrochloride [22,34,38]. Two of the studies specifically looked at postoperative pain following oncological surgeries and procedures with a one-time dose of 0.1 mg/kg ketamine administered perioperatively [22,38] and one study [34] examined the use of 0.2 mg/kg ketamine for visual analog scores > 3/10 over a 25 day period in refractory cancer pain therapy in combination with morphine to evaluate analgesic effects.

#### Effect on Pain Scores

Significant improvement in pain scores was found with administration of morphine in conjunction with intrathecal ketamine. One study found that a combination of bupivacaine, dexmedetomidine, and ketamine significantly improved postoperative analgesia when compared to either drug (dexmedetomidine or ketamine) alone [38]. In a similar surgical setting, a combination of intrathecal ketamine with morphine reduced total postoperative morphine consumption with good overall postoperative analgesia when compared to either drug alone [22]. Another study concluded that ketamine enhanced epidural morphine analgesia when administered in the early stages of terminal cancer pain therapy without increasing the incidence of adverse effects, while also reducing morphine requirement during the period of observation [34].

### 3.6. Intramuscular Administration of Ketamine

Four studies examined intramuscular administration of ketamine for the treatment of postoperative pain [23,31,40,41]. As seen in Table 2, one study examined a constant, fixed dose of ketamine [23], while the other compared escalating doses of intramuscular ketamine [41]. Preoperatively, a third study used 1 mg/kg ketamine in a Pecs block prior to breast cancer surgery [40]. Similarly, another study used either 0.5 mg/kg or 1 mg/kg of ketamine as part of a total peripheral nerve block in conjunction with bupivacaine [31].

#### Effect on Pain Scores

Two studies found that ketamine administration resulted in lower acute pain scores and morphine consumption following surgery [23,41]. Preoperatively, a modified Pecs block (ketamine + bupivacaine) prolonged the time to first request of analgesia and reduced total opioid consumption [40]. Ketamine, in addition to bupivacaine, as a peripheral nerve block preoperatively was also associated with lower morphine PCA consumption and longer analgesic effects [31].

### 3.7. Subcutaneous Infusion of Ketamine

One study examined the effects of subcutaneous infusions of ketamine as pain therapy for refractory cancer pain [29]. Ketamine alone was compared to placebo. As seen in Table 2, pain was evaluated with the Brief Pain Inventory score. The study utilized a dose-escalating regimen (100, 300, or 500 mg) of ketamine over a 5-day period in the treatment of refractory cancer pain.

#### Effect on Pain Scores

This study examining the effect of subcutaneous ketamine infusion on chronic cancer pain found that ketamine did not have a net clinical benefit when used as an adjunct to opioids and standard analgesics in refractory cancer pain [29].

### 3.8. Topical Administration of Ketamine

Two studies examined the topical administration of ketamine for the purpose of alleviating CIPN [24,28]. Similar dosages of ketamine (in addition to amitriptyline) were used in one study using up to 80mg of ketamine cream [28] compared to 20mg of ketamine applied twice daily in the other study [24].

#### Effect on Pain Scores

The study that utilized a greater dosage of ketamine suggested that two percent ketamine plus 4% amitriptyline cream does not decrease CIPN symptoms in cancer survivors [28]. Similarly, while pain scores improved following the administration of ketamine cream in the other study, the overall effect size was not large [24].

### 3.9. Oral Administration of Ketamine

Three studies assessed pain outcomes following oral administration of ketamine [27,30,35]. All studies examined either refractory oncogenic pain [35] or neuropathic pain [27,30]. Varied dosages of ketamine were used, as seen in Table 2.

#### Effect on Pain Scores

Pain scales varied, with one study using the VAS [35], and another using an index pain score from the sensory component of the short form McGill Pain Questionnaire [27]. While the studies examining neuropathic pain found no significant improvement in pain scores when compared to placebo [27,30], the study investigating refractory oncogenic pain found that ketamine was an effective co-adjuvant analgesic with morphine compared to morphine alone [35]. One study examining neuropathic pain noted the small number of patients studied, with only 22 patients analyzed following 6 drop-outs [30].

### 3.10. Adverse Effects of Ketamine

All studies assessed adverse side effects occurring following administration of ketamine, including psychiatric side effects or other adverse effects related to changes in blood pressure and respiratory, cardiovascular, or gastrointestinal changes. As can be seen in Table 3, side effects were assessed with a variety of different scales, including the Profile of Mood States (POMS) or through clinical signs such as heart rate. Most of the included studies reported minimal to no psychiatric side effects (such as dissociation, psychosis, or changes to cognition) with the administration of ketamine. One study found significant intergroup differences in the development of psychotoxicity following ketamine administration [29].

The most common adverse effects observed in the studies included nausea and vomiting [22,23,25,26,28,30,31,33,34,36,38,39,40,42]. In these studies, there were no significant differences between placebo and treatment groups in the incidence of gastrointestinal side effects such as nausea and vomiting. One study reported bladder spasms in 46.4% of patients in the treatment group; however, this effect was greatly reduced on postoperative days 1 and 2, with no differences being statistically significant [25]. Serious adverse effects, such as bradyarrhythmia [29,31,38] and cardiac arrest [29], were reported in some studies. Immediate postoperative sedation score was significantly increased in groups of patients administered ketamine compared to the control group in one study [38].

## 4. Discussion

To our knowledge, this is the first review focusing specifically on RCTs regarding the effectiveness of various forms of ketamine as an adjuvant to opioid therapy for managing acute and chronic pain among patients with cancer. Our results showed ketamine was most effective when used in conjunction with another analgesic such as morphine. When ketamine was used to reduce postoperative pain levels and morphine requirements postoperatively, it showed significant improvements in 13 of 14 studies [22,23,25,26,31,32,33,36,38,39,40,41,42]. However, ketamine was less effective when used as an analgesic for other types of pain arising from cancer, with four of the seven studies examining refractory cancer pain or neuropathic pain finding minimal effect on the reduction of pain scores or morphine requirements [27,28,29,30]. While multiple studies have shown that ketamine reduces refractory cancer pain [43,44,45], its use as a viable treatment option remains controversial. The difficulty in assessing effective pain control may lie in the heterogeneity of the cancer population and difficulty in defining outcomes in relation to pain. Psychological factors may also contribute to increased pain amongst patients with cancer, which might necessitate a more comprehensive approach than just the application of one intervention.

Our results showed that intravenous ketamine was most commonly used to reduce postoperative pain scores in the setting of acute pain rather than for refractory cancer pain. Ketamine was most often used preoperatively or intraoperatively via intravenous administration for postoperative pain control. Of the eight studies that examined postoperative pain control with intravenous ketamine, seven found that subanesthetic doses of ketamine significantly reduced pain outcomes or morphine consumption following surgery. Four of those studies used ketamine in conjunction with another analgesic agent such as morphine [25,33,39,42]. The use of ketamine as an opiate-sparing agent may be particularly important for patients who may have a tolerance to opiates. Patients with cancer and chronic cancer-related pain are more likely to have developed tolerance to opiates as a form of pain control [46]. Currently, the indications for esketamine, the “S” enantiomer form of ketamine, do not extend beyond treatment-resistant depression and suicidality [47]. Independent of long-term opioid therapy, depression is prevalent in 20–30% of patients with cancer [48]. Studies show that a bidirectional relationship may exist between depression and long-term opioid therapy in the treatment of non-cancer related pain [49,50,51,52]. While fewer studies have examined this relationship within the population of patients with cancer, the possible interdependence of depression and opioid use suggests a potential role for ketamine in addressing the difficulties of treating chronic cancer pain that may be refractory to opiate medications or neuropathic in nature. When considering the potential role of ketamine in chronic cancer pain management, the available data suggests that ketamine may be more efficacious when used in conjunction with an adjuvant analgesic. As a result, the introduction of novel analgesic agents such as ketamine may be integral in multimodal pain regimens for patients with cancer to address comorbidities such as depression and reduce requirements for opioid medications.

When used in combination with agents such as morphine as part of multimodal pain control, multiple studies demonstrated that ketamine is more likely to reduce pain scores and postoperative morphine consumption. This may be due to the fact that ketamine can attenuate morphine tolerance by increasing concentrations of morphine within the brain [53]. Ultimately, further research is needed to determine the degree to which the addition of ketamine to chronic cancer pain management may improve pain outcomes, along with consumption of oral morphine equivalents, throughout a patient’s experience with their disease.

This review showed that intrathecal administration of ketamine was most effective in reducing postoperative pain and terminal cancer pain in conjunction with other agents. The primary adjuvant agent administered with ketamine was morphine. Other agents utilized in conjunction with ketamine included bupivacaine and dexmedetomidine. These results are in line with previous research on intrathecal utilization of ketamine. In one meta-analysis of intrathecal administration of ketamine as an adjunct to bupivacaine following a variety of surgical procedures (including lower abdominal and lower limb surgery), time to first analgesic request was prolonged [54]. More studies are needed to determine the duration of the effects of ketamine following intrathecal administration. The benefits of intrathecal administration may be more prominent for postoperative pain outcomes rather than as an adjunct in treatment modalities for terminal cancer pain.

Administration of ketamine alone was most commonly performed in studies examining the intramuscular approach. Ketamine administration both before and after surgery was found to significantly reduce postoperative pain scores and delay rescue analgesia with morphine.

Included studies only examined oral administration in relation to pain outcomes associated with chronic cancer pain therapy. The highest dosage of ketamine of all 21 studies was utilized during oral administration, with up to 400 mg/day administered for patients [27]. However, this study did not find ketamine to have significantly greater effects than placebo, suggesting that escalating doses of oral ketamine are not effective for chronic cancer pain therapy. In addition, the efficacy of ketamine in this study was analyzed with respect to CIPN, which was the least investigated type of pain across all included studies. The lack of consistency in the assessment and diagnosis of CIPN may also make it difficult to assess for clinical improvements [7].

Finally, ketamine’s side effects, mainly neurological side effects, pose challenges to its utilization, emphasizing the importance of exploring alternative modes. Topical analgesics may play an important role in chronic pain management without the serious side effects associated with the medication. However, the two included studies [24,28] that evaluated topical ketamine use found that there was limited effect for CIPN. Of note, the daily dosages used for topical administration ranged from 40 mg to 80 mg. As no adverse systemic effects were reported at those doses in either study, further research on increased titration of ketamine within analgesic creams is warranted.

This review has several limitations. First, cancer-related pain may manifest differently, depending on the location of the tumor as well as the nature and severity of the cancer. In this aspect, the efficacy of ketamine in addressing pain may not be generalizable to all types and degrees of cancer. In addition, the analysis of pain, an already subjective measure, was investigated using a variety of scales across included studies. This could potentially influence the interpretation of the clinical efficacy of ketamine within our review. Second, in our assessment of the efficacy of ketamine for treatment of pain in cancer patients, we excluded studies that focused only on biological aspects of ketamine and thus may have missed papers that provided explanations of the molecular pathway between ketamine and pain. Finally, we may have missed relevant papers published in other languages by limiting our systematic review to English-only articles.

## 5. Conclusions

Intravenous ketamine, in dosages ranging from 0.1 mg/kg to 0.5 mg/kg, was most efficacious in improving pain scores in patients with cancer for up to 72 h following surgery, particularly in conjunction with other analgesics such as morphine. Fewer studies examined the use of ketamine for pain therapy, and those that did found less benefit in terms of pain scores following treatment for refractory chronic cancer pain (including CIPN). Ketamine was well tolerated across all studies that examined the side effects associated with ketamine administration.

## Figures and Tables

**Table 1:** Characteristics of the included studies.

Citation	Study Objective	Type of Cancer	Sample Size	Mean Age	Country	Setting
A. Abd El-Rahman et al. [23]	Compare the postoperative analgesic effect of local ketamine 1 mg/kg instilled in the wound to that of intramuscular ketamine and of placebo after total thyroidectomy.	Thyroid	90	43.4	Egypt	Inpatient
Abd El-Rahman, Mohamed et al. [22]	Investigate the effects of intrathecal morphine, ketamine, and their combination with bupivacaine for postoperative analgesia in major abdominal cancer surgery.	Abdominal	90	41.7	Egypt	Inpatient
Barton et al. [24]	Evaluate a topical baclofen, amitriptyline HCL, and ketamine in a pluronic lecithin organogel (BAK-PLO) to alleviate neuropathic pain, numbness, and/or tingling of chemotherapy-induced neuropathy (CIPN). Secondary goals included the evaluation of function, general pain, and toxicity.	N/A	208	61	USA	Outpatient
Chelly et al. [25]	Assess the effectiveness of a multimodal analgesic approach vs. patient-controlled analgesia (PCA) alone in patients undergoing open prostatectomy and assess the long-term benefit of our treatment modality.	Prostate	55	60	USA	Inpatient
de Kock et al. [26]	Investigate first whether ketamine has a specific effect on NMDA-related postoperative hyperalgesia and whether this drug could represent an efficient constituent of `balanced analgesia’. In addition, to determine ketamine’s preferential route of administration, either systemic or epidural.	Rectal adenocarcinoma	100	67	Belgium	Inpatient
Fallon et al. [27]	Comparison of oral ketamine with placebo for treating neuropathic pain in patients with cancer.	Mixed	214	58	UK	N/A
Gewandter et al. [28]	Investigate the efficacy of 2% ketamine plus 4% amitriptyline cream for reducing CIPN.	Mixed	462	N/A	USA	Outpatient
Hardy et al. [29]	Determine whether ketamine, delivered subcutaneously with dose titration over 5 days, has greater clinical benefit than placebo when used in conjunction with opioids and standard adjuvant therapy, in the management of chronic, uncontrolled pain related to cancer or its treatment.	Mixed	185	64	Australia	Inpatient
Ishizuka et al. [30]	The aim of this study was to evaluate the association of oral S(+) ketamine associated with morphine in controlling oncologic pain	Mixed	30	59	Brazil	Outpatient
Kamal et al. [31]	Investigate the effect of ketamine–bupivacaine in thoracic paravertebral block on acute and chronic pain after breast cancer surgery	Breast	90	49	Egypt	Inpatient
Kang et al. [32]	Test if intraoperative low-dose ketamine without postoperative infusion would reduce persistent postsurgical pain (PPSP) development after breast cancer surgery.	Breast	184	50.3	Korea	Outpatient
Kollender et al. [33]	Compare the effects of a standard morphine dose to a 35% lower dose plus a subanesthetic dose of ketamine for postoperative pain control in patients undergoing bone and soft tissue cancer surgery under standardized general anesthesia.	Bone and soft tissue cancer	60	41.5	Israel	Inpatient
Lauretti, Gomes et al. [34]	Examine analgesia and adverse effects of combination epidural pain therapy consisting of administration of morphine with either a low dose of ketamine, neostigmine, or midazolam in terminal cancer pain patients.	Mixed	48	53.8	Brazil	Inpatient
Lauretti, Lima et al. [35]	Evaluate the potential role of oral ketamine, an NMDA antagonist, or transdermal nitroglycerin, an NO donor, as coadjuvants to oral morphine in cancer pain therapy, compared with oral morphine alone or with the combination of a nonsteroidal anti-inflammatory drug (dipyrone) and oral morphine.	Mixed	60	55.3	Brazil	Inpatient
Lavand’homme et al. [36]	Examine the role and timing of balanced epidural analgesia as preventive treatment after major digestive surgery.	Rectal adenocarcinoma	85	53.4	Belgium	Inpatient/Outpatient
Mahran et al. [37]	Evaluate this assumption and compare the analgesic profile of preoperative pregabalin with ketamine in patients undergoing breast surgery.	Breast	90	53.5	Egypt	Inpatient
Mohamed et al. [38]	Investigate the efficacy and safety of intrathecal dexmedetomidine, ketamine, or both when added to bupivacaine for postoperative analgesia in major abdominal cancer surgeries.	Mixed	90	44.4	Egypt	Inpatient
Nesher et al. [39]	Assess if combining a subanesthetic dose of ketamine with morphine could effectively control pain while reducing postoperative morphine demand and drowsiness with an acceptable level of adverse side effects	Lung	41	59.5	Israel	Inpatient
Othman et al. [40]	Compare the analgesic efficacy and safety of modified Pecs block with ketamine plus bupivacaine versus bupivacaine in patients undergoing breast cancer surgery.	Breast	60	48.3	Egypt	Inpatient
Rakhman et al. [41]	Determine whether ketamine’s effect on acute postoperative pain could be enhanced and prolonged and analgesia consumption reduced if it was administered intramuscularly in repeated and escalating subanesthetic doses many hours before surgery.	Mixed	120	45.4	Israel	Inpatient
Shah et al. [42]	Compare two anesthetic techniques for modified radical mastectomy (MRM)—the conventional opioid-based technique versus an opioid-free and PECS-block-based technique.	Breast	70	51.7	India	Inpatient

**Table 2:** Ketamine administration details and its effectiveness on pain management based on route of administration.

Citation	Dosage of Ketamine (Including Duration)	How and When Was the Pain Measured?	Opioids Use and Dose	Does the Use of Ketamine Improve Pain Management?
Intramuscular				
Abd El-Rahman, El Sherif et al. [23]	1 mg/kg	Time to first request analgesia, Visual Analog Scale (VAS) at rest (VAS-R) and on movement (VAS-M) Measured immediately postoperatively, 1, 2, 4, 6, 12, and 24 h postoperatively	Induction of anesthesia included 2 mg/kg fentanyl. Postoperative analgesia comprised patient-controlled anesthesia with an initial morphine bolus of 0.1 mg/kg once pain was expressed by the patient or if the VAS was ≥3, followed by a 1 mg bolus with a 15-min lockout time.	Yes, local wound ketamine instillation provided superior postoperative analgesia with a lower incidence of side effects in comparison with intramuscular ketamine and placebo following total thyroidectomy.
Kamal et al. [31]	0.5 mg/kg ketamine (Group II) or 1 mg/kg ketamine (Group III)	Using a modified Observer’s Assessment of Alertness/Sedation scale (where 6 = agitated to 0 = does not respond to deep stimulus), VAS, time to first request of IV-PCA (which is defined as the time between the end of operation and tracheal extubation to the first request for supplemental analgesics and its administration to the patient), and the cumulative consumption of morphine PCA in the 1st 48 h postoperatively Measured at baseline (upon admission to the surgical intensive care unit (SICU)) and 2, 4, 6, 12, 24, 36, and 48h postoperatively	Fentanyl 50 μg, 100 mg morphine, and an initial morphine bolus of 0.1 mg/kg once pain was expressed by the patient or if the VAS score was ≥3, followed by a 1 mg bolus with a 15-min lockout time.	Yes, ketamine-bupivacaine in thoracic paravertebral block controlled acute postoperative pain in a dose-dependent manner and decreased DN4 scores one month after breast cancer surgery.
Othman et al. [40]	1 mg/kg	VAS Measured and assessed at baseline, one hour, 2 h, 4 h, 6 h, 12 h, 24 h, and 48 h postoperatively	Anesthesia was induced for all participating patients with 2 μg/kg fentanyl, 2–3 mg/kg propofol, and 1.5 mg/kg lidocaine.	Yes, the addition of ketamine to the modified Pecs block prolonged the time to the first request for analgesia and reduced total opioid consumption without serious side effects in patients who underwent a modified radical mastectomy.
Rakhman et al. [41]	1 dose (25 mg ketamine or 1 mL saline) at 4 h preoperatively (K1 or P1); 2 doses (10 and 25 mg ketamine or 1 mL saline twice) at 11 and 4 h (K2 or P2); or 3 doses (5, 10, and 25 mg ketamine or 1 mL saline thrice) at 17, 11, and 4 h preoperatively (K3 or P3).	Numerical rating scale (0–10) Measured every 15 min during the first postoperative hour and every 30 min until discharge from the PACU. On the ward, pain scores and vital signs were recorded every 6 h.	IV midazolam (2 mg), propofol (1–2.5 mg/kg), fentanyl (2–5 g/kg), and rocuronium or vecuronium to facilitate endotracheal intubation. Postoperatively, all patients received morphine (1.5 mg/bolus) via IV PCA. During patients’ stay in the PACU, 4 additional boluses of morphine could be added beyond the PCA protocol by the PACU anesthesiologist upon patient request; further requests to relieve pain were fulfilled with 75 mg of IM diclofenac.	Yes, repeated and escalating subanesthetic doses of intramuscular ketamine administered hours before surgery were associated with lower acute pain scores and lower IV PCA morphine consumption for 48 h after tumor surgery in the studied patients. These doses and the mode of delivery were well tolerated.
Intrathecal				
Abd El-Rahman, Mohamed et al. [22]	0.1 mg/kg ketamine in 1 mL volume	VAS Measured at 2, 4, 6, 12, 18, and 24 h postoperation	General anesthesia was induced with fentanyl 1.5 to 2 mg/kg. The morphine group received 10 mg of hyperbaric bupivacaine 0.5% in a 2 mL volume and 0.3 mg of morphine in a 1 mL volume intrathecally. The morphine + ketamine group received 0.3 mg of morphine. Rescue analgesia was represented by patient-controlled analgesia with intravenous morphine, with an initial bolus of 0.1 mg/kg	Yes, adding intrathecal ketamine 0.1 mg/kg to morphine 0.3 mg in patients who underwent major abdominal cancer surgery reduced the total postoperative morphine consumption in comparison with either drug alone, with overall good postoperative analgesia in all groups and no side effects apart from sedation.
Mohamed et al. [38]	0.1 mg/kg in a 1 mL volume	NRS, scored from 0–10 (where 0 = no pain and 10 = the worst pain imaginable) Measured immediately postoperatively, 2, 4, 6, 12, 18, and 24 h postoperatively	Intravenous morphine with an initial bolus of 0.1 mg/kg once pain was expressed by the patient, or if NRS was 3 or more (NRS ≥ 3). Total IV PCA morphine consumption (mg) in G1 was 9.16 ± 3.63, in G2 it was 8.66 ± 3.49, and in G3 it was 6.67 ± 2.8. General anesthesia was induced with fentanyl 1.5–2 µg/kg.	Yes, the combination of intrathecal dexmedetomidine and ketamine provided superior postoperative analgesia, prolonged the time to first request of rescue analgesia, and reduced the total consumption of PCA morphine without serious side effects compared to either drug alone.
Topical				
Barton et al. [24]	20 mg (twice a day, in the morning and before bed, for the duration of 4 weeks)	Quality of Life Questionnaire—Chemotherapy-Induced Peripheral Neuropathy (QLQCIPN20 or CIPN-20), European Organization for Research and Treatment of Cancer Quality of Life Questionnaire—CIPN20 (EORTC QLQ-CIPN20), Profile of Mood States (POMS), the Brief Pain Inventory (BPI), and the sensory neuropathy subsection of the NCI Common Terminology Criteria, version 3.0. Measured at baseline, before starting the study gel, and at 4 weeks	None	Yes, topical treatment with BAK-PLO appears to somewhat improve the symptoms of CIPN. This topical gel was well tolerated without evident systemic toxicity.
Gewandter et al. [28]	4g of KA cream (2% ketamine plus 4% amitriptyline) two times per day	NRS. Patients completed the seven-day daily pain, numbness, and tingling diary starting one week prior to entry into the study and at three and six weeks after study enrollment. The daily scores were averaged to calculate the pain, numbness, and tingling score for each data point. Measured 1 week prior to entry into study and 3 and 6 weeks after study enrollment	N/a	No, two percent ketamine plus 4% amitriptyline cream does not decrease CIPN symptoms in cancer survivors.
Oral				
Fallon et al. [27]	The starting dosage was 40mg/d, with a maximum dosage of 400 mg/d. Patients continued to receive a stable dosage for 16 days	Sensory Component of the Short Form, McGill Pain Questionnaire Measured at baseline and 16 days	The median morphine equivalent daily dose for both arms was 0 mg.	No, ketamine was equivalent to a placebo for cancer-related neuropathic pain.
Ishizuka et al. [30]	10 mg of ketamine	Pain severity was evaluated through a verbal scale, in which patients used the following scores: no pain = 0, mild = 1, moderate = 2, and severe = 3. Measured for four weeks, with interviews on the 7th, 14th, 21st, and 28th days	Oral morphine, 10 mg every 6 h, adjusted to every four hours if needed. The dose of morphine was increased (5 mg) whenever necessary, in each weekly evaluation, during the study.	No, a reduction in the need for opioids, lower pain scores, or greater pain relief in patients taking S(+) ketamine was not observed when compared to the placebo group, which goes against the reports in the literature for racemic ketamine.
Lauretti, Lima et al. [35]	0.5 mg/kg at 12 h intervals	VAS Measured on days 1, 5, 10, 15, 20, and 30 after the test drug was introduced	The morphine regimen was adjusted individually to a maximal oral dose of 80–90 mg/day to keep the VAS pain score less than 4. All patients in all groups had free access to as much morphine as they needed, with a maximum dose of 80–90 mg/day. At that point, when patients reported pain (VAS a-4), despite taking 80–90 mg of oral morphine daily, the test drug was added as follows: the control group received 20 mg of additional oral morphine (10 mg at 12 h intervals)	Yes, low-dose ketamine and transdermal nitroglycerin were effective co-adjuvant analgesics. In conjunction with their opioid tolerance-sparing function, joint delivery of ketamine or nitric oxide donors with opiates may be of significant benefit in cancer pain management.
Subcutaneous infusion				
Hardy et al. [29]	100, 300, or 500 mg were prepared by diluting ketamine hydrochloride 200 mg/2 mL in 24 h	BPI average pain score (reduction in BPI average pain score by greater than or equal to 2 points from baseline in the absence of more than four breakthrough doses of analgesia over the previous 24 h) Measured at the end of the 5-day study period	Minimum Daily Oral Dose of morphine, oxycodone, and hydromorphone was 60, 30, 8, and 20 mg, respectively. Minimum Parenteral Dose/24 Hours for morphine, oxycodone, hydromorphone, methadone, fentanyl, sufentanil, and alfentanil were 20 mg, 15 mg, 3 mg, 10 mg, 25 μg/h TTS or 600 μg SC/IV, 30 μg SC/IV, and 2 mg, respectively.	No, it had a strong placebo effect and failed to show any additional clinical benefit for ketamine when delivered subcutaneously in a dose-escalating regimen over 5 days, while significantly increasing toxicity.
Lauretti, Gomes et al. [34]	0.2 mg/kg epidural ketamine (2 mL)	VAS Measured within 25 days of observation (days 1, 2, 3, 8, 15, and 25)	The morphine regimen was adjusted individually to a maximal oral dose of 80–90 mg/day to keep the visual analog scale score less than 4. Pain was initially treated with epidural morphine 2 mg twice daily (12 hr intervals) to maintain the VAS below 4/10. Afterwards, VAS scores > 4/10 at any time were treated by adding the epidural study drug (2 mL), which was administered each morning, just after the 2 mg epidural morphine administration. The control group (CG) received 2 mg of epidural morphine (2 mL).	Yes, the association of low-dose epidural ketamine or neostigmine enhanced epidural morphine analgesia when administered in the early stages of terminal cancer pain therapy, without increasing the incidence of adverse effects, while ketamine also reduced the morphine requirement during the period of observation.
Intravenous				
Chelly et al. [25]	10 mg (1 mL)	Numeric Rating Scale (NRS) Measured every six hours (±two hours) until discharge, although the patients were not awakened for pain assessment during the night. Postoperative morphine consumption was also recorded at 24 hr and 48 hr (morphine equivalent)	Morphine 1–2 mg IV was given every ten minutes as needed to control immediate postoperative pain until the patient had free access to a morphine set-up delivering a morphine bolus of 1 mg with an eight-minute lockout	Yes, paravertebral blocks combined with celecoxib and ketamine provide better immediate postoperative pain control and facilitate earlier functional recovery in patients undergoing an open radical prostatectomy when compared with PCA alone.
De Kock et al. [26]	Ketamine at the bolus dose of 0.25 mg/kg, followed by an infusion of 0.125 mg/kg per h (group 2), 0.5 mg/kg and 0.25 mg/kg per h (group 3), epidural ketamine 0.25 mg/kg and 0.125 mg/kg per h (group 4), or 0.5 mg/kg and 0.25 mg/kg per h (group 5)	The cumulative number of met and unmet PCA morphine demands, the pain VAS scores at rest, at cough, and at mobilization, and the area of hyperalgesia for punctate mechanical stimuli around the surgical incision. Measured by the cumulative number of met and unmet PCA morphine demands at 2, 6, 12, 24, 36, and 48 h. The pain VAS scores were assessed by a blinded observer at 15 min, 2, 6, 12, 24, 36, and 48 h	All the patients, in any group considered, received an epidural bolus, including sufentanil 2.5 mg. This was immediately followed by an infusion that included sufentanil 0.75 mg/h at a rate of 4 ± 5 mL/h. This epidural infusion was stopped at the end of surgery.	Yes, subanesthetic doses of IV ketamine (0.5 mg/kg bolus followed by 0.25 mg/kg per h) given during anesthesia reduce wound hyperalgesia and are a useful adjuvant in perioperative balanced analgesia. Moreover, the systemic route is clearly the preferential route.
Kang et al. [32]	Between induction and skin incision, patients received a 0.25 mL/kg (0.5 mg/kg of ketamine or normal saline) study drug bolus followed by continuous infusion at 0.06 mL/kg/h (0.12 mg/kg of ketamine or normal saline) until the end of surgery	Via telephone, the first question was whether the patient had surgery-related pain. If the answer was positive, the investigator asked for their NRSr and NRSd questions from the Numeric Rating Scale for pain Measured 1, 3, and 6 months after surgery	Anesthesia was induced by total intravenous anesthesia using target-controlled infusion with propofol and remifentanil to reach 5 μg/mL and 4 ng/mL of effect site concentration (Ce), respectively. In order to ensure adequate and similar anesthesia between the groups, propofol was titrated to maintain a target Bispectral Index value between 40 and 50, and the remifentanil infusion rate was titrated between 2 and 4 ng/mL in order to keep mean arterial pressure within 20% of baseline. 0.1 mg/kg of morphine sulfate was administered along with 0.075 mg of palonosetron HCl. It contained morphine sulfate 100 mg (20 mL) with 80 mL of normal saline (1 mg/mL morphine sulfate). The pump delivered 2 mL boluses with a lockout period of 5 min and a 4 h limit of 20 mL. If the NRS-11 was more than 4 despite using the PCA, rescue medication (4 mg of morphine sulfate) was injected intravenously by a nurse in the postanesthetic care unit (PACU) and the general ward. The PCA was discontinued 72 h postoperatively.	Yes; while intraoperative low-dose ketamine without postoperative infusion significantly reduced the incidence of PPSP up to 3 months after breast cancer surgery, it failed to reduce clinically significant PPSP and improve patients’ quality of life.
Kollender et al. [33]	Drug injections consisted of a solution that contained 1.5 mg morphine (group MO) or 1 mg morphine plus 5 mg ketamine/bolus (group MK).	VAS Measured every 15 min for the first 2 h, every 30 min for the next 2 h and every 6 h until the IV-PCA device was disconnected	Drug injections consisted of a solution that contained 1.5 mg morphine (group MO) or 1 mg morphine plus the test drug (group MK).	Yes, the use of subanesthetic ketamine plus 2/3 of the standard dose of morphine following bone and tissue resections results in (1) a lower and more stable pain score, (2) a 60% morphine sparing effect, and (3) a shorter period of postoperative IV-PCA dependence.
Lavand’homme et al. [36]	0.5 mg/kg bolus followed by continuous infusion at 0.25 mg/kg/h) was started before skin incision and discontinued at the end of the procedure	VAS and patient-controlled analgesia Measured by the cumulative number of met PCA or PCEA demands at 12, 24, 48, and 72 h and visual analog scale pain scores at rest, cough, and mobilization assessed by a blinded observer at 30, 60, 90, and 120 min and 24, 48, and 72 h	Tracheal intubation was performed with 2.5 μg sufentanil and other anesthetics.	Yes, combined with an antihyperalgesic dose of ketamine, intraoperative epidural analgesia provides effective preventive analgesia after major digestive surgery.
Mahran et al. [37]	0.5 mg/kg ketamine in 5 mL of normal saline syringe IV before induction of anesthesia, followed by ketamine infusion at a rate of 0.25 mg/kg/h till the end of the surgery (the end of skin closure).	VAS at rest and with movement Measured after 30 min and subsequently after 2, 4, 6, 12, and 24 h	General anesthesia was induced with fentanyl 1–2 µg/kg IV. Additional doses of fentanyl were given so as to maintain HR within 15% of the baseline value and systolic arterial blood pressure within 20% of the baseline value. After emergence from anesthesia, the patients were transferred to the recovery room, and a PCA device was connected to the IV route of the patient. A solution of morphine (1 mg/mL) was prepared for the PCA. The PCA device was set for all groups with a demand dose of 1 mL and a lockout interval of 10 min, without a continuous background infusion.	No, neither the use of preoperative IV ketamine 0.5 mg/kg nor the preoperative oral use of 150 mg pregabalin could reduce VAS scores in patients undergoing breast cancer surgery, but they were proven in this study to reduce postoperative opioid requirements, rendering them a good co-analgesic in multi-modal analgesia with a good safety profile.
Nesher et al. [39]	1 mg of morphine plus a 5 mg ketamine bolus (MK).	VAS Measured every 15 min for 4 h	For anesthesia, 1 mg/kg; medium-dose fentanyl, patients were connected to patient-controlled IV analgesia, delivering 1.5 mg of morphine plus saline solution (MO) or 1.0 mg of morphine plus the test drug (MK). MO patients used 6.8 mg/h (mean) and 5.5 mg/h of morphine during the first and second hours, respectively; MK patients used 3.7 mg/h and 2.8 mg/h, respectively.	Yes, subanesthetic ketamine combined with a 35%-lower morphine dose provided equivalent pain control compared to the standard morphine dose alone, with fewer adverse side effects and a 45% reduction in morphine consumption.
Shah et al. [42]	Pre-incisional ketamine dose of 0.5 mg/kg, two more ketamine doses (0.25 mg/kg) were administered at 20-min intervals.	VAS Measured immediately after tracheal extubation and at one, two, four, eight, and 24 h postoperatively	Morphine 1.5 mg, fentanyl 2 µg/kg. Rescue analgesia comprised additional fentanyl boluses (20 µg each; maximum total dose 3 µg/kg) followed by 3 mg morphine boluses (maximum total dose 0.2 mg/kg) if VAS was still ≥3	Yes, pre-emptive PECS-blocks supplemented with low-dose ketamine and dexmedetomidine comprise a practical and useful alternative technique to the standard opioid-based general anesthetic technique for MRM.

**Table 3:** Secondary outcomes and side effects reported in included studies.

Citation	Secondary Outcome	How and When the Secondary Outcome Was Measured	Psychiatric (Dissociation, Psychosis, Cognitive, etc.) and Other (Hypo/Hypertension, Resp Depression, Cardiovascular Genitourinary, etc.) Adverse Side Effects
Abd El-Rahman, El Sherif et al. [23]	Side effects (nausea, vomiting, headache, chest pain, hallucination, delirium, arrhythmia)	Observed and recorded throughout the study period (24 h)	No psychiatric side effects. Other adverse effects included nausea, vomiting, and headache. Two patients developed hallucinations in group II (intramuscular ketamine); no significant difference was observed between the three groups regarding the incidence of side effects over the study period.
Abd El-Rahman, Mohamed et al. [22]	Vital signs	Measured by heart rate, noninvasive blood pressure, respiratory rate, and O_2_ saturation at 2, 4, 6, 12, 18, and 24 h postoperation	Dissociative effects and strange feelings were reported in the ketamine group. Other adverse effects included nausea, vomiting, nystagmus, dizziness, chest pain, dreams, and sedation.
Barton et al. [24]	Adverse events	Measured by the Profile of Mood States (POMS), the Brief Pain Inventory, and the sensory neuropathy subsection of the NCI Common Terminology Criteria, version 3.0, at baseline, before starting the study gel, and at 4 weeks	No psychiatric side effects. Other adverse effects included rash, constipation, dry mouth, confusion, and a depressed level of consciousness. No significant differences in toxicities were observed between the BAK arm and the placebo throughout the 4 weeks of the study.
Chelly et al. [25]	Functional status	Measured by SF-36 Health Survey (0–100) at 2, 4, 8, 12, and 24 weeks (±three days) by telephone interviews or during routine postoperative office visits.	No psychiatric side effects were assessed. Other adverse effects included: on the day of surgery, adverse effect bladder spasms were reported in 46.4% *(n* = 13) of patients in the MMA group compared with 40.7% (*n* = 13) of patients in the PCA group, and PONV was reported in 17.9% (*n* = 5) of patients in the MMA group compared with 18.5% (*n* = 5) of patients in the PCA group. On postoperative days one and two, the episodes of PONV and bladder spasms were greatly reduced; fewer of these episodes and less constipation in the MMA group than in the PCA group, although none of the differences were statistically significant.
De Kock et al. [26]	Residual pain	Patients were asked to answer the following questions. 1. Do you feel any pain at the scar area? If yes: do you take medication to alleviate it? If yes: do you take analgesic medications every day? And which one? Do you take analgesic medications occasionally (at least three times per week)? And which one? If no: do you feel particular sensations from the scar area? Itching, burning, sensibility? 2. Do you feel pain at any other place? If yes: where? Do you take analgesic medications? 3. Which unpleasant manifestations have you experienced since your operation? This inquiry was performed by phone and confirmed by mail. The incidence and importance of postoperative residual pain were evaluated at 2 weeks, 1 month, 6 months, and 1 year after surgery.	None of the considered patients experienced nightmares or psychotomimetic effects, whereas one patient in group 5 presented with hallucinations on the fourth postoperative day. No intergroup differences were noted in the results of the psychometric evaluations. For other adverse effects, the incidence of postoperative nausea was low in all the groups considered. Approximately 90% of patients presented with less than five episodes of nausea or vomiting during the 72 first postoperative hours.
Fallon et al. [27]	Mean and worst pain; mood	Hospital Anxiety and Depression Score, a self-administered anxiety and depression screening tool for use in nonpsychiatric patients. The tool has 14 items, which focus on the emotional and cognitive aspects of each aspect. Each item is scored from 0 to 3 for a combined maximum of 21 for each aspect, with higher scores reflecting a higher symptom load; mean change in global distress in the last 24 h	There were 18 serious adverse events: 8 in patients receiving ketamine and 10 in patients receiving placebo. Common adverse events were cognitive disturbance, dizziness, fatigue, nausea, and somnolence.
Gewandter et al. [28]	N/a	N/a	No psychiatric side effects were assessed. Adverse events (AEs) were assessed in the intent to treat population (*n* = 458). Two hundred ninety-five AEs were reported during the study; 147 occurred in the KA (ketamine) group and 158 occurred in the placebo group. Eight serious AEs were reported, with four in each arm. Twenty-one AEs were severe; ten occurred in the KA group and 11 in the placebo group. Five of the severe AEs were classified as musculoskeletal, two as swelling, and one as fatigue. The percent of subjects reporting AEs of all classes was similar between arms, although the study was not powered to detect differences in AEs.
Hardy et al. [29]	Pain assessments and adverse events	Graded according to the National Institutes of Health Common Terminology Criteria for Adverse Events, version 3.0. Psychomimetic-specific events were assessed daily using the Clinician Administered Dissociative States Scale (CADSS)	There was no difference in psychotoxicity at baseline, with approximately 40% of all participants having a positive CADSS score. Compared with the odds of the placebo group, the odds of the ketamine group experiencing psychotoxicity increased each day, becoming significant after day 3. For those with toxicity, when the level of toxicity between arms was compared, the ketamine group was more likely to report higher scores each day. By study end, the difference between groups was significant. There were relatively few adverse events higher than grade 3 in severity and worse than baseline (14 for ketamine; 16 for placebo). The most common were light-headedness (five cases), hypoxia (five cases), and somnolence (nine cases). Seven serious adverse events were reported, two of which (bradyarrhythmia and cardiac arrest, both in patients receiving ketamine) were thought to be possibly related to the study drug.
Ishizuka et al. [30]	N/a	N/a	The side effects observed did not show statistically significant differences between the groups, and constipation was reported by more than 60% of the patients in both groups. Nausea was present in 55% of the patients in G1 (morphine + ketamine) and in 30% of the patients in G2 (morphine alone), and vomiting was reported by 44% of the patients in G1 and in 23% of the patients in G2. Only three patients who took S(+) ketamine complained of sleepiness and delirium, but it did not prevent them from completing the study protocol.
Kamal et al. [31]	Chronic pain assessment	Measured by Douleur Neuropathique 4 (DN4) questions, assessed every month for the first three consecutive postoperative months	No psychiatric side effects were assessed. Perioperative adverse events were treated and recorded, such as nausea, vomiting, hypotension, hypertension, bradycardia, tachycardia, nystagmus, dizziness, emergence phenomenon, and sedation. There were no significant differences between the studied groups in the incidence of postoperative adverse effects or surgical complications.
Kang et al. [32]	Whether an abnormal sensation was present at the surgery site	Measured by the Douleur Neuropathique 4 (DN4) questionnaire at 1, 3, and 6 months after surgery	There were no differences in PONV, rescue medication, and the occurrence of psychotomimetic complications. Extubation time was longer, and shivering was less frequent in the ketamine group.
Kollender et al. [33]	Subjective sedation	Measured by VAS from 1 (fully awake) to 10 (heavily sedated) every 15 min for the first 2 h, every 30 min for the next 2 h, and every 6 h until the IV-PCA device was disconnected.	No psychiatric side effects were assessed. The MO (morphine group) patients’ rate of nausea and vomiting (PONV) was higher than that of the MK patients (morphine + ketamine group) (*p* < 0.05); all incidents were short-lived and responded well to metoclopramide. No ketamine-specific side effects were recorded; no patient of either group returned to the operating room for resurgery.
Lauretti, Gomes et al. [34]	Adverse effects assessment	Total number of patients complaining per total per group	There were no differences in adverse side effects among the groups. The only patient who suffered from hallucinations in the KG complained 28 days after the introduction of the study drug. Other adverse effects included somnolence, constipation, diminished appetite, skin redness/pain to epidural administration, back pain, nausea, or vomiting (no statistically significant differences among groups).
Lauretti, Lima et al. [35]	The daily consumption of morphine	Measured throughout the course of the study (30 days) on days 1, 5, 10, 15, 20, and 30 after the test drug was introduced	One patient from the KG (ketamine group) reported frequent hallucinations, and the oral dose was changed from 0.5 mg/kg to 0.25 mg/kg twice daily. One patient from the NG (nitroglycerin group) was withdrawn from the study because of an intense headache and was replaced by another patient to keep 15 patients in each group. Patients from the CG (control group) and the DG (dipyrone group) reported more somnolence compared with the KG and the NG (*p* < 0.013).
Lavand’homme et al. [36]	Residual Pain	The incidence and importance of postoperative residual pain were evaluated at 2 weeks and 1, 6, and 12 months after surgery by the following questions: 1. Do you feel any pain at the scar area? If yes: Do you take medication to alleviate it? Every day or occasionally (at least 2 times per week)? Which one(s)? If no: Do you have any particular sensations from the scar area? Itching, burning, sensitivity? 2. Do you feel pain at any other place? If yes: Where? Do you take analgesics? 3. Which unpleasant manifestations have you experienced since your operation? This inquiry was performed by the research nurse with a phone call and was confirmed by mail.	None of the considered patients experienced nightmares or psychomimetic effects. The incidence of postoperative nausea was low in all the groups considered. Orthostatic hypotension at first mobilization was significantly lower in patients receiving intravenous analgesia than in patients benefiting from epidural analgesia
Mahran et al. [37]	Sedation	Measured by a 4-point scale. (0—awake and alert, 1—mildly sedated, 2—moderately sedated, aroused by shaking, 3—deeply sedated, difficult to arouse even by shaking) in the first postoperative 24 h	Complications such as dizziness, visual disturbance, nightmares, and hallucinations were not recorded by any of the patients included in this study during the first 24 h of the postoperative period. No other adverse effects were assessed.
Mohamed et al. [38]	Postoperative adverse events	N/a	Nausea, vomiting, hypotension, bradycardia, cardiac arrhythmias, nystagmus, dissociative effects, strange feelings, dizziness, chest pain, dreams, and sedation. There was no significant difference among groups regarding postoperative sedation score except immediately postoperative, where there was a significant increase in sedation score in groups II (ketamine group) and III (dexmedetomidine + ketamine group) compared to group I (dexmedetomidine) (*p* = 0.02). There was a significant difference in the incidence of sedation (*p* < 0.03) in groups II and III compared to group I. Groups II and III had a higher incidence of sedation (3 [10.0%] and 5 [16.7%], respectively) compared to group I (0 [0.00%]). Apart from sedation, there were no significant differences in the incidence of other side effects between the 3 studied groups.
Nesher et al. [39]	Wakefulness	Measured by self-rated VAS, from 1 = heavily sedated to 10 = fully awake every 15 min for 4 h	One MK patient reported a sensation of lightheadedness that resolved spontaneously in <4 min, and at no time did any patients report hallucinations or postoperative confusion. The incidence of postoperative nausea and/or vomiting (PONV) was similar between the groups; all incidents were short-lived and responded well to appropriate therapy.
Othman et al. [40]	Hemodynamic variables, respiratory rate, oxygen saturation	Measured by systolic and diastolic blood pressure and heart rate, followed up and assessed at baseline, one hour, 2 h, 4 h, 6 h, 12 h, 24 h, and 48 h postoperatively	No psychiatric side effects were assessed. Other adverse effects included nausea and vomiting.
Rakhman et al. [41]	Analgesic consumption and the request rates of diclofenac	Measured hourly by PCA	No psychiatric side effects were assessed. Other adverse effects included dizziness and nausea.
Shah et al. [42]	Incidence of postoperative constipation, pruritus, PONV, HR, and MAP and time to discharge from SICU	Measured by clinical exam and patient interview at induction, intubation, surgical incision, every 15 min thereafter, end surgery, and one, two, four, eight, and 24 h postoperatively	No psychiatric side effects were assessed. Other adverse effects included nausea occurring immediately post tracheal extubation; nausea after receiving postoperative morphine for VAS ≥3; vomiting; constipation; pruritus, Sp02 ≤ 90%; RASS (Richmond agitation-sedation score). There was a higher incidence of PONV, constipation, xerostomia, and pruritus in Group O (opioids; sevoflurane).

## Data Availability

No new data were created or analyzed in this study. Data sharing is not applicable to this article.

## Appendix A. PRSMA 2020 Checklist: A Guideline for Reporting Systematic Reviews

**Section and Topic**	**Item #**	**Checklist Item**	**Location Where Item is Reported**
**TITLE**			
Title	1	Identify the report as a systematic review.	Page 1
**ABSTRACT**			
Abstract	2	See the PRISMA 2020 for Abstracts checklist.	Pages 2 and 3
**INTRODUCTION**			
Rationale	3	Describe the rationale for the review in the context of existing knowledge.	Pages 3 and 4
Objectives	4	Provide an explicit statement of the objective(s) or question(s) the review addresses.	Page 5
**METHODS**			
Eligibility criteria	5	Specify the inclusion and exclusion criteria for the review and how studies were grouped for the syntheses.	Page 5
Information sources	6	Specify all databases, registers, websites, organizations, reference lists, and other sources searched or consulted to identify studies. Specify the date when each source was last searched or consulted.	Pages 5 and 6
Search strategy	7	Present the full search strategies for all databases, registers, and websites, including any filters and limits used.	Pages 5 and 6
Selection process	8	Specify the methods used to decide whether a study met the inclusion criteria of the review, including how many reviewers screened each record and each report retrieved, whether they worked independently, and, if applicable, details of automation tools used in the process.	Page 6
Data collection process	9	Specify the methods used to collect data from reports, including how many reviewers collected data from each report, whether they worked independently, any processes for obtaining or confirming data from study investigators, and if applicable, details of automation tools used in the process.	Pages 6 and 7
Data items	10a	List and define all outcomes for which data were sought. Specify whether all results that were compatible with each outcome domain in each study were sought (e.g., for all measures, time points, and analyses), and if not, the methods used to decide which results to collect.	Pages 6 and 7
	10b	List and define all other variables for which data were sought (e.g., participant and intervention characteristics, and funding sources). Describe any assumptions made about any missing or unclear information.	Pages 6 and 7
Study risk of bias assessment	11	Specify the methods used to assess the risk of bias in the included studies, including details of the tool(s) used, how many reviewers assessed each study and whether they worked independently, and if applicable, details of automation tools used in the process.	Page 6
Effect measures	12	Specify for each outcome the effect measure(s) (e.g., risk ratio and mean difference) used in the synthesis or presentation of results.	NA
Synthesis methods	13a	Describe the processes used to decide which studies were eligible for each synthesis (e.g., tabulating the study intervention characteristics and comparing them against the planned groups for each synthesis (item #5)).	Pages 6 and 7
	13b	Describe any methods required to prepare the data for presentation or synthesis, such as handling missing summary statistics or data conversions.	Pages 6 and 7
	13c	Describe any methods used to tabulate or visually display the results of individual studies and syntheses.	Pages 6 and 7
	13d	Describe any methods used to synthesize results and provide a rationale for the choice(s). If meta-analysis was performed, describe the model(s), method(s) to identify the presence and extent of statistical heterogeneity, and software package(s) used.	NA
	13e	Describe any methods used to explore possible causes of heterogeneity among study results (e.g., subgroup analysis and meta-regression).	NA
	13f	Describe any sensitivity analyses conducted to assess the robustness of the synthesized results.	NA
Reporting bias assessments	14	Describe any methods used to assess the risk of bias due to missing results in a synthesis (arising from reporting biases).	NA
Certainty assessment	15	Describe any methods used to assess certainty (or confidence) in the body of evidence for an outcome.	NA
**RESULTS**			
Study selection	16a	Describe the results of the search and selection process, from the number of records identified in the search to the number of studies included in the review, ideally using a flow diagram.	Pages 7 and 8
	16b	Cite studies that might appear to meet the inclusion criteria but were excluded, and explain why they were excluded.	Pages 7 and 8
Study characteristics	17	Cite each included study and present its characteristics.	Page 8
Risk of bias in studies	18	Present assessments of the risk of bias for each included study.	Page 8
Results of individual studies	19	For all outcomes present in each study: (a) summary statistics for each group (where appropriate) and (b) an effect estimate and its precision (e.g., confidence/credible interval), ideally using structured tables or plots.	Pages 9–10
Results of syntheses	20a	For each synthesis, briefly summarize the characteristics and risk of bias among the contributing studies.	Page 9
	20b	Present the results of all statistical analyses conducted. If meta-analysis was done, present for each the summary estimate and its precision (e.g., confidence/credible interval) and measures of statistical heterogeneity. If comparing groups, describe the direction of the effect.	NA
	20c	Present the results of all investigations of possible causes of heterogeneity among study results.	NA
	20d	Present the results of all sensitivity analyses conducted to assess the robustness of the synthesized results.	NA
Reporting biases	21	Present assessments of the risk of bias due to missing results (arising from reporting biases) for each synthesis assessed.	Page 8
Certainty of evidence	22	Present assessments of certainty (or confidence) in the body of evidence for each outcome assessed.	NA
**DISCUSSION**			
Discussion	23a	Provide a general interpretation of the results in the context of other evidence.	Pages 9–13
	23b	Discuss any limitations of the evidence included in the review.	Page 14
	23c	Discuss any limitations of the review processes used.	Page 14
	23d	Discuss the implications of the results for practice, policy, and future research.	Pages 9–13
**OTHER INFORMATION**			
Registration and protocol	24a	Provide registration information for the review, including the register name and registration number, or state that the review was not registered.	Page 5
	24b	Indicate where the review protocol can be accessed, or state that a protocol was not prepared.	Page 5
	24c	Describe and explain any amendments to the information provided at registration or in the protocol.	Pages 5 to 9
Support	25	Describe the sources of financial or non-financial support for the review and the role of the funders or sponsors in the review.	Page 14
Competing interests	26	Declare any competing interests of the review authors.	Page 15
Availability of data, code, and other materials	27	Report which of the following are publicly available and where they can be found: template data collection forms; data extracted from included studies; data used for all analyses; analytic code; any other materials used in the review.	Page 5
		From: Page MJ et al. [55]. For more information, visit: http://www.prisma-statement.org/ (accessed on 22 February 2022).	

## Appendix B. Inclusion and Exclusion Criteria for Screening Studies

**Study Characteristic**	**Inclusion Criteria**	**Exclusion Criteria**
Population	- Adult patients with cancer	- Children (defined as individuals less than 18 years old)
Intervention	- Ketamine administration via oral, IV, IM, subcutaneous, intrathecal, or topical routes	
Comparison	- Patients with cancer who did not receive ketamine	
Outcome	- Pain	- Any outcomes not listed
Study design	- Randomized trials - Nonrandomized trials - Controlled before-after studies - Cross-sectional - Qualitative studies - Interrupted time-series studies or repeated measures studies - Prospective and retrospective observational studies (i.e., cohort studies and case control studies)	- Descriptive studies with no outcome data - Case reports and case studies - Modeling studies that used simulated data - Not a clinical study (e.g., editorial, nonsystematic review, and letter to the editor) - Measurement or validation studies - Self-described pilot studies without adequate power to assess the impact of interventions on outcomes.
Time/location	- No limitation	
Publication types	- Full publication in a peer-reviewed journal - Dissertations	- Meeting abstracts, protocols without results, and gray literature

## Appendix C. Search Strategy Sample

- Source PubMed
  Items found 773
  25 Mar 2024
  Search Query

(((Ketamine[Mesh] OR Ketamin*[ TIAB] OR Ketalar[TIAB] OR Ketaject[TIAB] OR Ketanest[TIAB] OR esketamin*[ TIAB] OR spravato*[TIAB])) AND ((Neoplasms[mesh] OR neoplasms[tiab] OR neoplasm[tiab] OR neoplasia[tiab] OR neoplasias[tiab] OR neoplastic[tiab] OR dysplastic[tiab] OR dysplasia[tiab] OR dysplasias[tiab] OR “Early Detection of Cancer”[Mesh] OR cancer[tiab] OR cancers[tiab] OR cancerous[tiab] OR malignant[tiab] OR malignancy[tiab] OR malignancies[tiab] OR metastatic[tiab] OR metastasis[tiab] OR metastases[tiab] OR “Biomarkers, Tumor”[Mesh] OR tumor[tiab] OR tumors[tiab] OR tumour[tiab] OR tumours[tiab] OR adenocarcinoma[tiab] OR adenocarcinomas[tiab] OR carcinoma[tiab] OR carcinomas[tiab] OR sarcoma[tiab] OR sarcomas[tiab] OR lymphoma[tiab] OR lymphomas[tiab] OR melanoma[tiab] OR melanomas[tiab] OR leukemia[tiab] OR leukemias[tiab] OR “Cancer Care Facilities”[Mesh] OR “Oncology Service, Hospital”[Mesh] OR oncology[tiab] OR oncologic[tiab] OR chemotherapy[tiab] OR chemotherapies[tiab] OR “neoadjuvant therapy”[tiab] OR “neoadjuvant therapies”[tiab] OR chemoradiotherapy[tiab] OR chemoradiotherapies[tiab] OR radioimmunotherapy[tiab] OR radiotherapy[tiab] OR radioimmunotherapies[tiab])) AND (english[Filter])) NOT ((“infant”[mesh] OR “child”[mesh] OR “adolescent”[mesh] OR infant[tiab] OR child[tiab] OR adolescent[tiab] OR teenager[tiab] OR Juvenile[tiab]) AND (english[Filter])) AND (english[Filter]) AND (english[Filter])

Source Embase
Items found 3260
25 Mar 2024
Search Query

(‘esketamine’/exp OR ‘ketamine’/exp OR ‘ketofol’/exp OR ketamin*:ab,ti OR ketalar:ab,ti OR ketaject:ab,ti OR ketanest:ab,ti OR esketamin*:ab,ti OR ketofol*:ab,ti OR arketamine*:ab,ti OR ketalar*:ab,ti OR spravato*:ab,ti) AND (‘malignant neoplasm’/exp OR ‘metastasis’/exp OR ‘lymphoma’/exp OR ‘sarcoma’/exp OR ‘chemoradiotherapy’/exp OR ‘chemotherapy’/exp OR ‘oncology ward’/exp OR neoplasms:ab,ti OR neoplasm:ab,ti OR neoplasia:ab,ti OR neoplasias:ab,ti OR neoplastic:ab,ti OR dysplastic:ab,ti OR dysplasia:ab,ti OR dysplasias:ab,ti OR cancer:ab,ti OR cancers:ab,ti OR cancerous:ab,ti OR malignant:ab,ti OR malignancy:ab,ti OR malignancies:ab,ti OR metastatic:ab,ti OR metastasis:ab,ti OR metastases:ab,ti OR tumor:ab,ti OR tumors:ab,ti OR tumour:ab,ti OR tumours:ab,ti OR adenocarcinoma:ab,ti OR adenocarcinomas:ab,ti OR carcinoma:ab,ti OR carcinomas:ab,ti OR sarcoma:ab,ti OR sarcomas:ab,ti OR lymphoma:ab,ti OR lymphomas:ab,ti OR melanoma:ab,ti OR melanomas:ab,ti OR leukemia:ab,ti OR leukemias:ab,ti OR oncology:ab,ti OR oncologic:ab,ti OR chemotherapy:ab,ti OR chemotherapies:ab,ti OR ‘neoadjuvant therapy’:ab,ti OR ‘neoadjuvant therapies’:ab,ti OR chemoradiotherapy:ab,ti OR chemoradiotherapies:ab,ti OR radioimmunotherapy:ab,ti OR radiotherapy:ab,ti OR radioimmunotherapies:ab,ti) NOT (infant:ab,ti OR child:ab,ti OR adolescent:ab,ti OR teenager:ab,ti OR juvenile:ab,ti OR ‘infant’/exp OR ‘child’/exp OR ‘adolescent’/exp) AND [english]/lim

(ketamin*:ab,ti OR ketalar:ab,ti OR ketaject:ab,ti OR ketanest:ab,ti OR esketamin*:ab,ti OR ketofol*:ab,ti OR arketamine*:ab,ti OR ketalar*:ab,ti OR spravato*:ab,ti) AND (‘malignant neoplasm’/exp OR ‘metastasis’/exp OR ‘lymphoma’/exp OR ‘sarcoma’/exp OR ‘chemoradiotherapy’/exp OR ‘chemotherapy’/exp OR ‘oncology ward’/exp OR neoplasms:ab,ti OR neoplasm:ab,ti OR neoplasia:ab,ti OR neoplasias:ab,ti OR neoplastic:ab,ti OR dysplastic:ab,ti OR dysplasia:ab,ti OR dysplasias:ab,ti OR cancer:ab,ti OR cancers:ab,ti OR cancerous:ab,ti OR malignant:ab,ti OR malignancy:ab,ti OR malignancies:ab,ti OR metastatic:ab,ti OR metastasis:ab,ti OR metastases:ab,ti OR tumor:ab,ti OR tumors:ab,ti OR tumour:ab,ti OR tumours:ab,ti OR adenocarcinoma:ab,ti OR adenocarcinomas:ab,ti OR carcinoma:ab,ti OR carcinomas:ab,ti OR sarcoma:ab,ti OR sarcomas:ab,ti OR lymphoma:ab,ti OR lymphomas:ab,ti OR melanoma:ab,ti OR melanomas:ab,ti OR leukemia:ab,ti OR leukemias:ab,ti OR oncology:ab,ti OR oncologic:ab,ti OR chemotherapy:ab,ti OR chemotherapies:ab,ti OR ‘neoadjuvant therapy’:ab,ti OR ‘neoadjuvant therapies’:ab,ti OR chemoradiotherapy:ab,ti OR chemoradiotherapies:ab,ti OR radioimmunotherapy:ab,ti OR radiotherapy:ab,ti OR radioimmunotherapies:ab,ti) NOT (infant:ab,ti OR child:ab,ti OR adolescent:ab,ti OR teenager:ab,ti OR juvenile:ab,ti OR ‘infant’/exp OR ‘child’/exp OR ‘adolescent’/exp) AND [english]/lim

Source Scopus
Items found 141
25 Mar 2024
Search Query

(TITLE-ABS (ketamin* OR ketalar OR ketaject OR ketanest AND esketamin* OR ketamin* OR ketalar OR ketaject OR ketanest OR ketofol* OR arketamine* OR ketalar* OR spravato*) AND TITLE ABS (neoplasms OR neoplasm OR neoplasia OR neoplasias OR neoplastic OR dysplastic OR dysplasia OR dysplasias OR cancer OR cancers OR cancerous OR malignant OR malignancy OR malignancies OR metastatic OR metastasis OR metastases OR tumor OR tumors OR tumour OR tumours OR adenocarcinoma OR adenocarcinomas OR carcinoma OR carcinomas OR sarcoma OR sarcomas OR lymphoma OR lymphomas OR melanoma OR melanomas OR leukemia OR leukemias OR oncology OR oncologic OR chemotherapy OR chemotherapies OR neoadjuvant AND therapy OR neoadjuvant AND therapies OR chemoradiotherapy OR chemoradiotherapies OR radioimmunotherapy OR radiotherapy OR radioimmunotherapies) AND NOT TITLE-ABS-KEY (infant OR child OR adolescent OR juvenile OR teenager))

## Appendix D. Quality Assessment Tools Used for Assessing the Quality of Included Studies

NIH Quality Assessment Tool and Ratings for the Controlled Intervention Studies

	**Abd El-Rahman et al. (2017)** [22]	**Abd El-Rahman et al.** **(2018)** [23]	**Barton et al. (2011)** [24]	**Chelly et al. (2011)** [25]	**De Kock et al. (2001)** [26]	**Fallon et al. (2018)** [27]	**Gewandter et al. (2014)** [28]	**Hardy et al. (2012)** [29]	**Ishizuka et al. (30)**	**Kamal et al. (2019)** [31]	**Kang et al. (2020)** [32]	**Kollender et al. (2008)** [33]	**Lauretti, Gomes, et al. (1999)** [34]	**Lauretti, Lima, et al. (1999)** [35]	**Lavand’homme et al. (2005)** [36]	**Mahran et al. (2015)** [37]	**Mohamed et al. (2016)** [38]	**Nesher et al. (2009)** [39]	**Othman et al. (2016)** [40]	**Rakhman et al. (2011)** [41]	**Shah et al. (2020)** [42]
Was the study described as randomized, a randomized trial, a randomized clinical trial, or an RCT?	Yes	Yes	Yes	Yes	Yes	Yes	Yes	Yes	Yes	Yes	Yes	Yes	Yes	Yes	Yes	Yes	Yes	Yes	Yes	Yes	Yes
Was the method of randomization adequate (i.e., use of randomly generated assignments)?	Yes	Yes	Yes	Yes	Yes	Yes	Yes	Yes	Yes	Yes	Yes	Yes	Yes	Yes	Yes	Yes	Yes	Yes	Yes	Yes	Yes
Was the treatment allocation concealed (so that assignments could not be predicted)?	Yes	Yes	Yes	Yes	Yes	Yes	Yes	Yes	Yes	Yes	Yes	Yes	Yes	Yes	Yes	Yes	Yes	Yes	Yes	Yes	Yes
Were study participants and providers blinded to treatment group assignment?	Yes	Yes	Yes	Yes	Yes	Yes	Yes	Yes	Yes	Yes	Yes	Yes	Yes	Yes	Yes	Yes	Yes	Yes	Yes	Yes	Yes
Were the people assessing the outcomes blinded to the participants’ group assignments?	Yes	Yes	Yes	Yes	Yes	Yes	Yes	Yes	Yes	Yes	Yes	Yes	Yes	No	Yes	Yes	Yes	Yes	Yes	Yes	Yes
Were the groups similar at baseline on important characteristics that could affect outcomes (e.g., demographics, risk factors, co-morbid conditions)?	Yes	Yes	Yes	Yes	Yes	Yes	Yes	Yes	Yes	Yes	Yes	Yes	Yes	Yes	Yes	Yes	Yes	Yes	Yes	Yes	Yes
Was the overall drop-out rate from the study at the endpoint 20% or lower than the number allocated to treatment?	Yes	Yes	No	Yes	Yes	No	Yes	Yes	Yes	Yes	Yes	Yes	Yes	Yes	Yes	Yes	Yes	Yes	Yes	Yes	Yes
Was the differential drop-out rate (between treatment groups) at the endpoint 15 percentage points or lower?	Yes	Yes	Yes	Yes	Yes	Yes	Yes	Yes	Yes	Yes	Yes	Yes	Yes	Yes	Yes	Yes	Yes	Yes	Yes	Yes	Yes
Was there high adherence to the intervention protocols for each treatment group?	Yes	Yes	Yes	Yes	Yes	Yes	Yes	Yes	Yes	Yes	Yes	Yes	Yes	Yes	Yes	Yes	Yes	Yes	Yes	Yes	Yes
Were other interventions avoided or similar in the groups (e.g., similar background treatments)?	Yes	Yes	Yes	Yes	Yes	Yes	Yes	Yes	Yes	Yes	Yes	Yes	Yes	Yes	Yes	Yes	Yes	Yes	Yes	Yes	Yes
Were outcomes assessed using valid and reliable measures, implemented consistently across all study participants?	Yes	Yes	Yes	Yes	Yes	Yes	Yes	Yes	Yes	Yes	Yes	Yes	Yes	Yes	Yes	Yes	Yes	Yes	Yes	Yes	Yes
Did the authors report that the sample size was sufficiently large to be able to detect a difference in the main outcome between groups with at least 80% power?	Yes	Yes	Yes	No	Yes	Yes	Yes	No	No	Yes	Yes	Yes	Yes	Yes	Yes	Yes	Yes	Yes	Yes	Yes	Yes
Were outcomes reported or subgroups analyzed prespecified (i.e., identified before analyses were conducted)?	Yes	Yes	Yes	Yes	Yes	Yes	Yes	Yes	Yes	Yes	Yes	Yes	Yes	Yes	Yes	Yes	Yes	Yes	Yes	Yes	Yes
Were all randomized participants analyzed in the group to which they were originally assigned? (i.e., did they use an intention-to-treat analysis?)	Yes	Yes	Yes	Yes	Yes	Yes	Yes	Yes	Yes	Yes	Yes	Yes	Yes	Yes	Yes	Yes	Yes	Yes	Yes	Yes	Yes
Overall quality rating	Good	Good	Good	Good	Good	Good	Good	Good	Good	Good	Good	Good	Good	Good	Good	Good	Good	Good	Good	Good	Good
			Overall quality: Good, Fair, Poor.																		
