# Correction: Azari et al. The Efficacy of Ketamine for Acute and Chronic Pain in Patients with Cancer: A Systematic Review of Randomized Controlled Trials. *Healthcare* 2024, *12*, 1560

**DOI:** 10.3390/healthcare12222219

**Published:** 2024-11-07

**Authors:** Leila Azari, Homa Hemati, Ronia Tavasolian, Sareh Shahdab, Stephanie M. Tomlinson, Margarita Bobonis Babilonia, Jeffrey Huang, Danielle B. Tometich, Kea Turner, Kimia Saleh Anaraki, Heather S. L. Jim, Amir Alishahi Tabriz

**Affiliations:** 1 Morsani College of Medicine, University of South Florida, Tampa, FL 33602, USA; smtomlinson@usf.edu; 2 College of Pharmacy, Tehran University of Medical Sciences, Tehran 1416753955, Iran; hemati.homa98@gmail.com (H.H.); shahdabsareh@gmail.com (S.S.); 3 Department of Clinical Science and Nutrition, University of Chester, Chester CH1 4BJ, UK; ronia_tsn@yahoo.com; 4 Supportive Care Medicine Department, Behavioral Medicine Services, Moffitt Cancer Center, Tampa, FL 33612, USA; margarita.bobonis-babilonia@va.gov; 5 Department of Psychiatry and Behavioral Medicine, University of South Florida Morsani College of Medicine, Tampa, FL 33602, USA; 6 Department of Oncological Sciences, University of South Florida Morsani College of Medicine, Tampa, FL 33602, USA; kea.turner@moffitt.org (K.T.); heather.jim@moffitt.org (H.S.L.J.); amir.alishahi@moffitt.org (A.A.T.); 7 Department of Anesthesiology, Moffitt Cancer Center, Tampa, FL 33612, USA; jeffrey.huang@moffitt.org; 8 Department of Health Outcomes and Behavior, Moffitt Cancer Center, Tampa, FL 33612, USA; danielle.tometich@moffitt.org; 9 Department of Internal Medicine, University of Maryland Capital Region, Largo, MD 20774, USA; kimia.salehanaraki@umm.edu

## Error in Appendix

In the original publication [1], there was a mistake in “*Appendix C. Search Strategy Sample*” as published. While the search strategy is correct, the number of studies before and after duplicate removal should be corrected. The corrected “*Appendix C. Search Strategy Sample*” appears below. The authors state that the scientific conclusions are unaffected. This correction was approved by the Academic Editor. The original publication has also been updated.

## Appendix C. Search Strategy Sample

- Source PubMed
  Items found 773
  25 Mar 2024
  Search Query

(((Ketamine[Mesh] OR Ketamin*[ TIAB] OR Ketalar[TIAB] OR Ketaject[TIAB] OR Ketanest[TIAB] OR esketamin*[ TIAB] OR spravato*[TIAB])) AND ((Neoplasms[mesh] OR neoplasms[tiab] OR neoplasm[tiab] OR neoplasia[tiab] OR neoplasias[tiab] OR neoplastic[tiab] OR dysplastic[tiab] OR dysplasia[tiab] OR dysplasias[tiab] OR “Early Detection of Cancer”[Mesh] OR cancer[tiab] OR cancers[tiab] OR cancerous[tiab] OR malignant[tiab] OR malignancy[tiab] OR malignancies[tiab] OR metastatic[tiab] OR metastasis[tiab] OR metastases[tiab] OR “Biomarkers, Tumor”[Mesh] OR tumor[tiab] OR tumors[tiab] OR tumour[tiab] OR tumours[tiab] OR adenocarcinoma[tiab] OR adenocarcinomas[tiab] OR carcinoma[tiab] OR carcinomas[tiab] OR sarcoma[tiab] OR sarcomas[tiab] OR lymphoma[tiab] OR lymphomas[tiab] OR melanoma[tiab] OR melanomas[tiab] OR leukemia[tiab] OR leukemias[tiab] OR “Cancer Care Facilities”[Mesh] OR “Oncology Service, Hospital”[Mesh] OR oncology[tiab] OR oncologic[tiab] OR chemotherapy[tiab] OR chemotherapies[tiab] OR “neoadjuvant therapy”[tiab] OR “neoadjuvant therapies”[tiab] OR chemoradiotherapy[tiab] OR chemoradiotherapies[tiab] OR radioimmunotherapy[tiab] OR radiotherapy[tiab] OR radioimmunotherapies[tiab])) AND (english[Filter])) NOT ((“infant”[mesh] OR “child”[mesh] OR “adolescent”[mesh] OR infant[tiab] OR child[tiab] OR adolescent[tiab] OR teenager[tiab] OR Juvenile[tiab]) AND (english[Filter])) AND (english[Filter]) AND (english[Filter])

Source Embase
Items found 3260
25 Mar 2024
Search Query

(‘esketamine’/exp OR ‘ketamine’/exp OR ‘ketofol’/exp OR ketamin*:ab,ti OR ketalar:ab,ti OR ketaject:ab,ti OR ketanest:ab,ti OR esketamin*:ab,ti OR ketofol*:ab,ti OR arketamine*:ab,ti OR ketalar*:ab,ti OR spravato*:ab,ti) AND (‘malignant neoplasm’/exp OR ‘metastasis’/exp OR ‘lymphoma’/exp OR ‘sarcoma’/exp OR ‘chemoradiotherapy’/exp OR ‘chemotherapy’/exp OR ‘oncology ward’/exp OR neoplasms:ab,ti OR neoplasm:ab,ti OR neoplasia:ab,ti OR neoplasias:ab,ti OR neoplastic:ab,ti OR dysplastic:ab,ti OR dysplasia:ab,ti OR dysplasias:ab,ti OR cancer:ab,ti OR cancers:ab,ti OR cancerous:ab,ti OR malignant:ab,ti OR malignancy:ab,ti OR malignancies:ab,ti OR metastatic:ab,ti OR metastasis:ab,ti OR metastases:ab,ti OR tumor:ab,ti OR tumors:ab,ti OR tumour:ab,ti OR tumours:ab,ti OR adenocarcinoma:ab,ti OR adenocarcinomas:ab,ti OR carcinoma:ab,ti OR carcinomas:ab,ti OR sarcoma:ab,ti OR sarcomas:ab,ti OR lymphoma:ab,ti OR lymphomas:ab,ti OR melanoma:ab,ti OR melanomas:ab,ti OR leukemia:ab,ti OR leukemias:ab,ti OR oncology:ab,ti OR oncologic:ab,ti OR chemotherapy:ab,ti OR chemotherapies:ab,ti OR ‘neoadjuvant therapy’:ab,ti OR ‘neoadjuvant therapies’:ab,ti OR chemoradiotherapy:ab,ti OR chemoradiotherapies:ab,ti OR radioimmunotherapy:ab,ti OR radiotherapy:ab,ti OR radioimmunotherapies:ab,ti) NOT (infant:ab,ti OR child:ab,ti OR adolescent:ab,ti OR teenager:ab,ti OR juvenile:ab,ti OR ‘infant’/exp OR ‘child’/exp OR ‘adolescent’/exp) AND [english]/lim

(ketamin*:ab,ti OR ketalar:ab,ti OR ketaject:ab,ti OR ketanest:ab,ti OR esketamin*:ab,ti OR ketofol*:ab,ti OR arketamine*:ab,ti OR ketalar*:ab,ti OR spravato*:ab,ti) AND (‘malignant neoplasm’/exp OR ‘metastasis’/exp OR ‘lymphoma’/exp OR ‘sarcoma’/exp OR ‘chemoradiotherapy’/exp OR ‘chemotherapy’/exp OR ‘oncology ward’/exp OR neoplasms:ab,ti OR neoplasm:ab,ti OR neoplasia:ab,ti OR neoplasias:ab,ti OR neoplastic:ab,ti OR dysplastic:ab,ti OR dysplasia:ab,ti OR dysplasias:ab,ti OR cancer:ab,ti OR cancers:ab,ti OR cancerous:ab,ti OR malignant:ab,ti OR malignancy:ab,ti OR malignancies:ab,ti OR metastatic:ab,ti OR metastasis:ab,ti OR metastases:ab,ti OR tumor:ab,ti OR tumors:ab,ti OR tumour:ab,ti OR tumours:ab,ti OR adenocarcinoma:ab,ti OR adenocarcinomas:ab,ti OR carcinoma:ab,ti OR carcinomas:ab,ti OR sarcoma:ab,ti OR sarcomas:ab,ti OR lymphoma:ab,ti OR lymphomas:ab,ti OR melanoma:ab,ti OR melanomas:ab,ti OR leukemia:ab,ti OR leukemias:ab,ti OR oncology:ab,ti OR oncologic:ab,ti OR chemotherapy:ab,ti OR chemotherapies:ab,ti OR ‘neoadjuvant therapy’:ab,ti OR ‘neoadjuvant therapies’:ab,ti OR chemoradiotherapy:ab,ti OR chemoradiotherapies:ab,ti OR radioimmunotherapy:ab,ti OR radiotherapy:ab,ti OR radioimmunotherapies:ab,ti) NOT (infant:ab,ti OR child:ab,ti OR adolescent:ab,ti OR teenager:ab,ti OR juvenile:ab,ti OR ‘infant’/exp OR ‘child’/exp OR ‘adolescent’/exp) AND [english]/lim

Source Scopus
Items found 141
25 Mar 2024
Search Query

(TITLE-ABS (ketamin* OR ketalar OR ketaject OR ketanest AND esketamin* OR ketamin* OR ketalar OR ketaject OR ketanest OR ketofol* OR arketamine* OR ketalar* OR spravato*) AND TITLE ABS (neoplasms OR neoplasm OR neoplasia OR neoplasias OR neoplastic OR dysplastic OR dysplasia OR dysplasias OR cancer OR cancers OR cancerous OR malignant OR malignancy OR malignancies OR metastatic OR metastasis OR metastases OR tumor OR tumors OR tumour OR tumours OR adenocarcinoma OR adenocarcinomas OR carcinoma OR carcinomas OR sarcoma OR sarcomas OR lymphoma OR lymphomas OR melanoma OR melanomas OR leukemia OR leukemias OR oncology OR oncologic OR chemotherapy OR chemotherapies OR neoadjuvant AND therapy OR neoadjuvant AND therapies OR chemoradiotherapy OR chemoradiotherapies OR radioimmunotherapy OR radiotherapy OR radioimmunotherapies) AND NOT TITLE-ABS-KEY (infant OR child OR adolescent OR juvenile OR teenager))
